# Optimal timing of induction of labour to improve maternal and perinatal outcomes: protocol for an individual participant data and network meta-analysis

**DOI:** 10.1136/bmjopen-2025-112155

**Published:** 2026-01-08

**Authors:** Hollie Meacham, Abraham Ona-Igbru, Rachel McNeill, Ruth Ajayi, Emily Pickering, William A Grobman, Mairead Black, Asma Khalil, Christine Mccourt, Angela Miranda, Ben W Mol, Kate Walker, Amie Wilson, Javier Zamora, Shakila Thangaratinam, John Allotey

**Affiliations:** 1 Department of Women’s and Children’s Health, University of Liverpool Institute of Life Course and Medical Sciences, Liverpool, England, UK; 2 Patient and Public Involvement and Engagement Representative, Birmingham, UK; 3 Division of Maternal Fetal Medicine, Brown University Warren Alpert Medical School, Providence, Rhode Island, USA; 4 Care New England Health System, Providence, Rhode Island, USA; 5 Aberdeen Centre for Women’s Health Research, University of Aberdeen, Aberdeen, Scotland, UK; 6 Fetal Medicine Unit, St George’s University of London, London, England, UK; 7 Centre for Maternal and Child Health Research, University of London, London, UK; 8 City St George’s University of London, London, England, UK; 9 Clinical Biostatistics Unit, Hospital Universitario Ramón y Cajal, Madrid, Spain; 10 Department of Obstetrics and Gynaecology, Monash University, Melbourne, Victoria, Australia; 11 Centre for Perinatal Research, University of Nottingham School of Medicine, Nottingham, England, UK; 12 Department of International Public Health, Liverpool School of Tropical Medicine, Liverpool, England, UK; 13 Liverpool Women’s NHS Foundation Trust, Liverpool, England, UK; 14 NIHR Applied Research Collaboration North West Coast, Liverpool, England, UK

**Keywords:** Pregnancy, OBSTETRICS, Meta-Analysis, Network Meta-Analysis, Mortality, Pregnant Women

## Abstract

**Abstract:**

**Introduction:**

Despite advances in maternity care, stillbirth remains a major burden. It disproportionately affects black and Asian mothers, those with obesity and women over the age of 35 years. Induction of labour may benefit these women, but there is no clear evidence to guide recommendations on optimal timing of induction because of variations in the intervention and insufficient power in primary trials for rare outcomes such as stillbirth and perinatal mortality, or to assess whether effects differ by maternal characteristics. We will conduct an individual participant data (IPD) meta-analysis of randomised trials to assess the overall and differential effect of induction of labour, according to timing of induction and maternal characteristics, on adverse perinatal and maternal outcomes. We will also rank induction of labour timing strategies by their effectiveness to inform clinical and policy decision-making.

**Methods and analysis:**

We will identify randomised trials on induction of labour by searching MEDLINE, CINAHL, EMBASE, BIOSIS, LILACS, Pascal, SCI, CDSR, ClinicalTrials.gov, ICTRP, ISRCTN registry, CENTRAL, DARE and Health Technology Assessment Database, without language restrictions, from inception to June 2025. Primary researchers of identified trials will be invited to join the OPTIMAL Collaboration and share the original trial data. Data integrity and trustworthiness assessment will be performed on all eligible trials. We will check each study’s IPD for consistency with the original authors before standardising and harmonising the data. Study quality of included trials will be assessed by the Cochrane Risk of Bias tool. We will perform a series of one-and-two-stage random-effects meta-analyses to obtain the summary intervention effect on composite adverse perinatal outcome (stillbirth, neonatal death or severe morbidity requiring admission to neonatal unit) with 95% CIs and summary treatment–covariate interactions (maternal age, ethnicity, parity, socioeconomic status, body mass index and method of conception). Heterogeneity will be summarised using tau^2^, I^2^ and 95% prediction intervals for effect in a new study. Sensitivity analysis to explore robustness of statistical and clinical assumptions will be carried out. Small study effects (potential publication bias) will be investigated using funnel plots.

**Ethics and dissemination:**

The study is registered on PROSPERO (CRD420251066346) and ethics approval is not required. We will disseminate findings widely to women, healthcare professionals and policymakers through academic, professional bodies and social media channels, and in peer-reviewed journals to achieve impact.

**PROSPERO registration number:**

CRD420251066346.

**STRENGTHS AND LIMITATIONS OF THIS STUDY**

- An individual participant data (IPD) meta-analysis will provide greater power than aggregate data meta-analysis to detect differential effects of the timing of induction of labour across maternal subgroups (eg, age, ethnicity, parity, socioeconomic status, body mass index and method of conception).
- Access to raw data allows for precise assessment of optimal gestational age to reduce adverse perinatal outcomes and analysis of subgroup even when such variables were not reported in the original publication.
- Use of IPD enables network meta-analysis to compare timing strategies, while adjusting for effect modifiers to reduce any inconsistency in the network.
- Limitations include potential lack of access to all trial data and reliance on investigators’ willingness to prepare and share data sets, which may reduce the number of studies available compared with aggregate data meta-analysis.

## Background

 The UK has one of the highest stillbirth rates among high-income countries,^1^ and perinatal mortality rates are rising after 7 years of gradual decline,^2^ with wide disparities by ethnicity and social deprivation. Stillbirth rates increased by 6% in white women and 17% in black women,^2^ and babies born to black women now have the highest rates of stillbirth and neonatal death. In the UK, black women are 117% and Asian women 57% more likely to suffer a stillbirth than white women,^1^ with risks threefold higher in obese women,^3^ twofold higher in mothers from the most deprived areas and 1.5-fold to twofold higher in mothers aged ≥35 years.^2 4^ Addressing these inequities is central to the UK Government’s 2023 National Health Service (NHS) mandate on providing high-quality equitable care,^5^ as bereaved mothers are four to seven times more likely to experience depression,^6^ recurrent pregnancy loss,^7^ long-term health disorders^8^ and marital breakdown, with the effects extending to partners and siblings.^9^

The risk of adverse perinatal outcomes rises with each additional week beyond term (>37 weeks’ gestation),^10^ and pregnancies continuing past the estimated due date carry an increased risk of stillbirth and neonatal mortality.^10^ About a third of stillbirths happen at term in mothers without known medical complications,^11^ with risks increasing steeply after 41 weeks’ gestation.^10^ In women who are older, obese, black or Asian, or who conceived through fertility treatment, stillbirth risk begins to rise from as early as 39 weeks.^11^ Induction of labour remains one of the few interventions that can reduce stillbirth rates,^12^ and identifying which women would benefit most remains a priority.^13^

Despite many randomised trials to date on induction of labour,^14^ there is no clear evidence to guide recommendations on optimal timing of induction for rare but serious outcomes such as stillbirth and perinatal mortality. Individual studies lack sufficient power to show a reduction in stillbirth alone, by timing or within clinically relevant subgroups of women, and evidence from aggregate meta-analyses is limited by ecological bias, lack of participant-level data and variation in induction timing across trials. These uncertainties contribute to inconsistent clinical decision-making and may lead to unnecessary inductions, which can negatively affect birth experiences and informed maternal choice.

We plan to undertake an individual participant data (IPD) meta-analysis of induction of labour to evaluate its effects on adverse perinatal and maternal outcomes, and whether these vary by maternal characteristics including age, ethnicity, parity, socioeconomic status, body mass index and method of conception, as well as by intervention characteristics. We will use network meta-analysis techniques to compare the effectiveness of different induction timing strategies and identify the optimal gestational age to prevent adverse perinatal outcomes.

### Objectives

Primary

To determine using IPD meta-analysis of randomised trials,

1. The overall effect of induction of labour compared with expectant management or delayed induction, on composite adverse perinatal outcome (stillbirth, neonatal death and severe neonatal morbidity requiring admission to the neonatal unit).
2. The differential effect of induction of labour according to intervention (induction of labour timing strategies) and maternal (age, ethnicity, parity, socioeconomic status, body mass index and method of conception) characteristics on composite adverse perinatal outcome.

Secondary

1. To evaluate the effect of the intervention on critically important individual maternal and perinatal outcomes.
2. To undertake an IPD network meta-analysis to produce a rank order by effectiveness of induction of labour timing strategies.
3. To assess the association between specific gestational age of induction and key pregnancy outcomes to identify potential thresholds for optimal timing of induction of labour.

## Methods

The protocol adheres to Preferred Reporting Items for Systematic review and Meta-Analysis Protocols (PRISMA-P) reporting statement^15^ and has been registered on The International Prospective Register of Systematic reviews (PROSPERO) with registration ID: CRD420251066346. Our IPD meta-analytical approach will follow existing methodological guidelines and adhere to the PRISMA-IPD reporting statement.^16^

### Patient and public involvement

Patients with lived experiences of induction of labour and stillbirth have been involved with this work throughout and have informed design, outcome selection and source of funding. They will be involved in all stages of the project, including reporting of findings.

### Literature search

We will update the literature search up to June 2025 using our existing search strategy,^14^ to identify new trials (published and unpublished) since the completion of our previous review. We will search databases such as MEDLINE, CINAHL, EMBASE, BIOSIS, LILACS, Pascal, SCI, CDSR, ClinicalTrials.gov, ICTRP, ISRCTN registry, CENTRAL, DARE and Health Technology Assessment Database using search terms for the intervention such as ‘labour, induced, cervical ripening’, combined with terms for ‘expectant management, conservative management, watchful waiting, prolonged, post-term, post-date, full-term and 37–45 week’ and randomised trials. See online supplemental appendix 1 for the full search strategy and search terms. The searches will also be expanded to include a manual search of relevant reference lists from included studies. Language restrictions will not be applied.

### Eligibility criteria

We will include randomised (individual or cluster) trials at or beyond 37 weeks’ gestation that compare (1) planned induction of labour at a specified gestational age versus expectant management or (2) two or more planned induction timings. Animal studies, non-experimental studies and cross-over trials will be excluded.

### Outcome measures

Our primary outcome is a composite adverse perinatal outcome of stillbirth, neonatal death or severe morbidity requiring admission to neonatal unit, informed by the core outcome set for randomised trials on induction of labour.^17^ We chose the composite outcome and its components for the following reasons: death of a baby, as stillbirth or perinatal death, is equally important. However, given the rarity of stillbirth, we will have insufficient power (<5%) to detect treatment effects and differences across subgroups. We included admission to the neonatal unit, as it is considered a critically important core outcome by healthcare professionals and women, with potential long-term implications for baby and additional treatment costs.^13 17^ A term baby of an uncomplicated pregnancy is rarely expected to be admitted to the neonatal unit; such an admission may be indicative of significant neonatal morbidity. Inclusion of this component also addresses concerns about competing risks between components of the composite, where earlier induction reduces stillbirth but increases neonatal complications.

Our secondary outcomes include maternal and neonatal complications such as maternal death, uterine rupture, caesarean section, instrumental birth, postpartum haemorrhage, intensive care unit admission, blood transfusion, shoulder dystocia, neonatal hypoglycaemia and neonatal seizures (table 1). Subgroup analysis will explore whether a woman’s age, ethnicity, parity, socioeconomic status, body mass index and method of conception modify the intervention effect.

**Table 1:** Structured research question

Question components	Description
Population	Unselected pregnant women with no medical obstetric indication for induction of labour
Intervention	Induction of labour at or beyond 37 weeks’ gestation (as intention to treat and as received)
Comparison	Expectant management/delayed induction
Outcomes	Primary outcomes Composite of adverse perinatal outcome (stillbirth, neonatal death or severe morbidity requiring admission to neonatal unit) Secondary outcomes Maternal: maternal death, uterine rupture, caesarean section, postpartum haemorrhage, instrumental birth, third/fourth degree perineal tear, pre-eclampsia, chorioamnionitis, length of hospital stay, ICU admission, blood transfusion, duration in labour, postnatal depression/anxiety/trauma, measures of childbirth experience and satisfaction with birth, epidural usage Offspring: stillbirth, neonatal death, admission to NICU, any neonatal admission, length of stay, hypoglycaemia, hyperbilirubinaemia, shoulder dystocia, transfusion of blood products, hypoxic ischaemic encephalopathy or need for therapeutic hypothermia, Apgar ≤3 at 5, meconium aspiration syndrome, seizure, birth trauma, respiratory support, infection, birth weight below 2500 g
Study design	Randomised trials

ICU, intensive care unit; NICU, neonatal intensive care unit.

### Study selection

Two researchers will independently select studies using a two-stage process. The first stage will involve title and abstract screening, followed by assessment of full texts of selected studies in detail to confirm eligibility. Any disagreements will be resolved via consensus or consultation of a third reviewer. We will then contact authors of eligible studies, requesting their raw IPD which will then be standardised and cleaned. Study level data extraction will be done in duplicates and will include country, setting, inclusion and exclusion criteria of participants, details of the intervention and control, primary trial aim and definition and assessment of the primary outcome.

### Establishment of the OPTIMAL collaborative network

We will establish a collaborative network of investigators involved in the primary induction of labour studies by contacting relevant researchers of the eligible trials via email, inviting them to join the network and share their IPD. We currently have agreement in principle from six researchers to share data from their trials (12 013 women), making up >70% of participants recruited in induction of labour trials since 2000 (online supplemental appendix 2). A bespoke database will be developed for collaborators to supply data and authors will be encouraged to share their data in whichever format is convenient for them. Once received, we will convert all data into a standardised format following our established and trialled procedures used in our previous IPD meta-analysis for data extraction, formatting, cleaning and harmonisation.^18 19^ This will facilitate smooth and timely execution of the most time-consuming stage of the study.

### Quality assessment

The quality of the IPD from each study will be assessed independently by two researchers using the Cochrane ROB 2 tool for assessing risk of bias in randomised trials.^20^ We will conduct sensitivity analyses to examine the robustness of statistical and clinical conclusions to inform the inclusion or exclusion of trials considered to be at high risk of bias.

### Data and integrity checks

The TRACT (Trustworthiness in Randomised Controlled Trials) tool^21^ and the IPD Integrity tool^22^ will be used to conduct integrity checks for each IPD received. These tools aim to identify and triage randomised trials at risk of trustworthiness or data integrity issues and involve reviewing the completeness and accuracy of the data. Any inconsistencies such as missing data or extreme values (discrepancies between raw and published data) will be resolved with the original author. All communications resolving these discrepancies will be recorded.

### Sample size consideration

Formal sample size calculations are not usually undertaken for meta-analyses. The statistical power for our meta-analysis is dependent on the available IPD. We expect to have a sample size of at least 12 013 women from the trials that have already agreed to share data in principle. The sample size has the potential to increase further depending on decisions to share data by other identified studies.

Based on previous reviews,^14 23^ we expect the magnitude of effect of induction of labour compared with expectant management to be 40% reduction in odds of perinatal death. With a 5.4% average baseline risk of composite outcome in the expectant management group, we will have 80% power with 3000 participants to detect a statistically significant reduction. Assuming a similar magnitude of difference between subgroups, as a rule of thumb, the sample size needed will be at least four times that needed for the main effect.^24^ In this case, 12 000 participants will be enough to detect significant interactions of the same magnitude as the main effect.

We have undertaken a simulation-based approach to calculate the power of estimating genuine treatment–covariate interactions in our planned IPD meta-analysis, conditional on sample size of 12 000 participants and assuming our sample size increases to 14 000. The results are shown in table 2 below for an assumed interaction between covariate and treatment effect for a 40% (OR 0.60) and 50% (OR 0.50) reduction in composite outcome, based on risk-factor prevalence and variations in the subgroup sizes ratio. With our current agreed sample size of 12 000 women, we have 50–72% power (depending on prevalence of covariate). If the sample size is increased to 14 000 women, based on awaited responses, we estimate the power to increase to 82% for genuine treatment–covariate interactions for a 40% reduction in our composite outcome.

**Table 2:** Estimated power for treatment–covariate interaction by simulation

Risk factor prevalence	Subgroup ratio	Estimated power by simulation for the six trials agreed to share IPD (n=12 000)	Estimated power by simulation assuming half of IPD from studies not yet agreed to share data do in addition to the six trials already agreed (n=1400)
Assuming an interaction between covariate and treatment effect that corresponds to an OR of 0.60			
50%	1.1	72%	82.4%
33.3%	1.2	65%	71.5%
25%	1.3	56.3%	65.1%
20%	1.4	49%	59.0%
Assuming an interaction between covariate and treatment effect that corresponds to an OR of 0.50			
50%	1.1	91%	95.1%
33.3%	1.2	86%	92.0%
25%	1.3	78.9%	86.9%
20%	1.4	71%	77.0%

IPD, individual participant data.

### Data analysis

#### Overall effect and by timing strategies

The effectiveness of induction of labour will be assessed using the IPD meta-analytical framework^25^ and will be based on IPD studies meeting the trustworthiness criteria. For the overall effect and by different timing strategies beyond 37 weeks’, we will perform one-stage and two-stage IPD random effect meta-analyses fitted using frequentist (Restricted Maximum Likelihood (REML) with Hartung-Knapp correction) or Bayesian (with vague or empirically derived prior distributions) methods, to obtain the pooled intervention effect on composite adverse perinatal outcome. CIs will be inflated to account for uncertainty in variance estimates (using Hartung-Knapp and Kenward-Roger corrections). Heterogeneity will be summarised using I^2^ statistic and the estimated between-study variance (‘tau-squared’). We will calculate a 95% prediction interval for the intervention effect when applied in an individual clinical setting. The above analyses will also be undertaken for secondary critically important individual maternal and perinatal outcomes.

#### Differential effect by subgroups (treatment–covariate interactions)

We will examine whether the women’s age, ethnicity, parity, socioeconomic status, body mass index and conception method are factors that modify the effect of different induction of labour timing strategies. This will be undertaken by extending the two-stage and one-stage meta-analysis framework to include and then summarise treatment–covariate interaction terms, which provides the change in intervention effect for a 1-unit change in the covariate. Continuous variables will be kept as continuous to avoid arbitrary dichotomisation, and non-linear relationships and interactions modelled using splines and fractional polynomials. Subgroup analyses, if not carefully planned, can lead to misleading results (due to the play of chance with multiple testing).^26^ Thus, caution will be used in interpreting the collective set of subgroup results, and adjustment for multiple testing will be considered as necessary. However, we reiterate here that our IPD meta-analysis will increase the power (often >80%) to detect genuine subgroup effects and will also allow us to examine if there is consistency in the subgroup effect from study to study, rather than being a chance finding in a single study, for example.

#### IPD network meta-analysis

We will perform an IPD network meta-analysis to compare and rank intervention effects for various induction of labour timing strategies using direct and indirect comparisons, adjusting for covariates that modify treatment effect to alleviate any inconsistency in the network.^27 28^ A multivariate adjustment for maternal characteristics will ensure consistency of indirect comparisons of the network meta-analysis because women randomised in trials are induced at different gestational ages, so the comparison needs to be adjusted to account for these differences. Summary results and evidence of consistency before and after covariate adjustment will be provided.

#### Examining potential sources of bias

Small study effects (potential publication bias) will be investigated using funnel plots. To examine the impact of non-IPD studies, we will extract and incorporate aggregate data that meets trustworthiness criteria alongside the IPD within the two-stage IPD random-effect meta-analysis if appropriate.

#### Dealing with missing variables

A range of strategies will be considered for dealing with missing data in covariates. For randomised trials, mean imputation or the missing indicator method is appropriate to handle missing data in covariates.^29^ If necessary, we will use multiple imputations to impute partially missing variables within each study separately, under a missing at random assumption. Systematically missing variables, where considered plausible, will be imputed by borrowing information across studies, allowing for heterogeneity and clustering in a multilevel imputation model.^30^ Other missing variables could even be calculated using other pieces of raw data. For example, even if data on body mass index were not published, we could generate this if raw data on weight and height were available.

## Ethics and dissemination

The IPD meta-analysis will not require ethics approval as it is an evidence synthesis involving meta-analysis of anonymised data sets. Our findings will be provided as a detailed report to the funder, presented at conferences (nationally and internationally) and shared with policy makers, guideline developers and key stakeholders as an infographic and at organised workshop events. We will liaise with them to ensure that updates to existing recommendations, guidelines and policies are timetabled to incorporate our findings. We will share our findings with women and their support networks via established charity organisations with the help of our advisory group.

## Discussion

We propose an IPD meta-analysis of randomised trials, where the raw participant-level data are obtained and synthesised across trials, to determine the effects of induction of labour on adverse perinatal outcomes and whether these vary by maternal and intervention characteristics. We will use IPD network meta-analysis techniques to compare the effectiveness of different induction of labour timing strategies and determine the optimal gestational age of induction to prevent adverse perinatal outcomes.

Reducing stillbirth and neonatal death remains a national priority, supported by the Secretary of State, the Royal College of Obstetricians and Gynaecologists and NHS England.^31^,^33^ In 2018, the total health and social care cost of stillbirth in the UK was estimated as £13.6 million each year, funeral-related expenses cost parents £1.8 million, litigation costs £2.5 million, productivity losses of parents and healthcare professionals were £12.5 million, while productivity loss of the child was £693.7 million.^34^ Any intervention that reduces stillbirths therefore has great potential to provide major clinical, economic and societal benefit.

Although the 2020 Cochrane review suggested induction of labour from 37 weeks’ reduces perinatal death and other adverse outcomes compared with expectant management,^23^ limitations in study power, imprecision in gestational age comparisons and suboptimal subgroup analyses prevented NICE (National Institute for Health and Care Excellence) from making high-quality recommendations on optimal timing of induction of labour. Our proposed IPD meta-analysis will have greater power than aggregate meta-analysis to detect differential treatment effects, as it can model individual risk status (prognostic factor) across participants within trials, thus explaining variability in outcomes and identifying subgroups that benefit most.^35^ Our findings will directly inform policy and practice through future iterations of the NHS Saving Babies Lives Care Bundle and NICE Induction of Labour guidance, and will also help optimise use of resources by avoiding unnecessary interventions and targeting induction to women most likely to benefit.

Limitations include potential difficulties in accessing IPD from all eligible trials due to lack of contact with original trial authors, unwillingness to share data or because primary data sets are no longer accessible. These will be transparently reported in our PRISMA flow diagram, and sensitivity analyses will assess the impact of including aggregate data that meets trustworthiness criteria to the IPD studies. Variations in how individual level variables are reported across trials may limit our ability to fully explore subgroup effects or treatment–covariate interactions. To minimise this limitation, we will follow robust data cleaning and harmonisation procedures to maximise comparability and reliability of analyses.

By reusing existing trial data, this project represents a cost-efficient approach that minimises research waste and delivers answers more quickly than new large-scale trials.

## Supplementary material

10.1136/bmjopen-2025-112155 online supplemental file 1
