# Supplementary material for: Optimal timing of induction of labour to improve maternal and perinatal outcomes: protocol for an individual participant data and network meta-analysis
Source: BMJ Open. 2026 Jan 8;16(1):e112155. doi: 10.1136/bmjopen-2025-112155 (PMC13059840; doi:10.1136/bmjopen-2025-112155)
Supplement: online supplemental file 1 [file bmjopen-16-1-s001.pdf]

Appendices

Appendix 1. Search strategy used for the systematic review

Cochrane Central Register of Controlled Trials (CENTRAL)

Query	Search
#1	(((induced and (labor or labour)) or induction or (expectant and management))) ti,ab,kw
#2	full-term or fullterm or post-date or postdate or post-term or postterm or prolonged or protracted or postmature or post-mature or "late term" or "beyond term" or "37* week*" or "38* week*" or "39* week*" or "40* week*" or "41* week*" or "42* week*" or "43* week*" or "44* week*" or "45* week*" or prolonged) NEAR/4 (pregnancy or pregnancies or delivery or deliveries)) ti,ab,kw
#3	#1 AND #2
#4	[mh ^"LABOR, INDUCED"]
#5	(((labor or labour) near/5 induc*)):ti,ab,kw (Word variations have been searched)
#6	[mh ^"CERVICAL RIPENING"]
#7	((cervi* near/3 ripen*)):ti,ab,kw (Word variations have been searched)
#8	(((unfavorabl* or unfavourabl* or un-favorabl* or un-favourabl* or unripe* or un-ripe*) near/3 cervi*)):ti,ab,kw (Word variations have been searched)
#9	(((bishop* or cerv*) near/3 scor*)):ti,ab,kw (Word variations have been searched)
#10	#4or#5or#6or#7or#8or#9
#11	[mh ^"CONSERVATIVE TREATMENT"]
#12	((conservative* near/3 (manag* or treat* or policy or policies))):ti,ab,kw (Word variations have been searched)
#13	((expect* near/3 manag*)):ti,ab,kw (Word variations have been searched)
#14	[mh ^"WATCHFUL WAITING"]
#15	((watchful* near/3 wait*)):ti,ab,kw (Word variations have been searched)
#16	(("no treat*" or "non treat*")):ti,ab,kw (Word variations have been searched)
#17	(("no interven*" or "non interven*")):ti,ab,kw (Word variations have been searched)
#18	(("no induc*" or "non induc*")):ti,ab,kw (Word variations have been searched)

#19 (spontaneous* near/5 (labor or labour or deliver* or onset or "follow* up"))

#20 (((fetal or foetal or fetus*or foetus) near/5 (test* or monitor*))):ti,ab,kw (Word variations have been searched)

#21 #11or#12or#13or#14or#15or#16or#17or#18or#19or#20

#22 [mh ^"PREGNANCY, PROLONGED"]

#23 (((prolonged or protracted or postmature or post-mature or postterm or post- term or postdate* or post-date*) near/5 pregnanc*)):ti,ab,kw (Word variations have been searched)

#24 ("full term"):ti,ab,kw (Word variations have been searched)

#25 #22 or #23 or #24

#26 ("37* week*"):ti,ab,kw (Word variations have been searched)

#27 ("38* week*"):ti,ab,kw (Word variations have been searched)

#28 ("39* week*"):ti,ab,kw (Word variations have been searched)

#29 ("40* week*"):ti,ab,kw (Word variations have been searched)

#30 ("41* week*"):ti,ab,kw (Word variations have been searched)

#31 ("42* week*"):ti,ab,kw (Word variations have been searched)

#32 ("43* week*"):ti,ab,kw (Word variations have been searched)

#33 ("44* week*"):ti,ab,kw (Word variations have been searched)

#34 ("45* week*"):ti,ab,kw (Word variations have been searched)

#35 #26or#27or#28or#29or#30or#31or#32or#33or#34

#36 (("37* week*" near/5 ("38* week*" or "39* week*" or "40* week*" or "41* week*" or "42* week*" or "43* week*" or "44* week*" or "45* week*"))):ti,ab,kw (Word variations have been searched)

#37 (("38* week*" near/5 ("37* week*" or "39* week*" or "40* week*" or "41* week*" or "42* week*" or "43* week*" or "44* week*" or "45* week*"))):ti,ab,kw (Word variations have been searched)

#38 (("39* week*" near/5 ("37* week*" or "38* week*" or "40* week*" or "41* week*" or "42* week*" or "43* week*" or "44* week*" or "45* week*"))):ti,ab,kw (Word variations have been searched)

#39 (("40* week*" near/5 ("37* week*" or "38* week*" or "39* week*" or "41* week*" or "42* week*" or "43* week*" or "44* week*" or "45* week*"))):ti,ab,kw (Word variations have been searched)

#40 (("41* week*" near/5 ("37* week*" or "38* week*" or "39* week*" or "40* week*" or "42* week*" or "43* week*" or "44* week*" or "45* week*"))):ti,ab,kw (Word variations have been searched)

#41 (("42* week*" near/5 ("37* week*" or "38* week*" or "39* week*" or "40* week*" or "41* week*" or "43* week*" or "44* week*" or "45* week*"))):ti,ab,kw (Word variations have been searched)

#42 (("43* week*" near/5 ("37* week*" or "38* week*" or "39* week*" or "40* week*" or "41* week*" or "42* week*" or "44* week*" or "45* week*"))):ti,ab,kw (Word variations have been searched)

#43 (("44* week*" near/5 ("37* week*" or "38* week*" or "39* week*" or "40* week*" or "41* week*" or "42* week*" or "43* week*" or "45* week*"))):ti,ab,kw (Word variations have been searched)

#44 (("45* week*" near/5 ("37* week*" or "38* week*" or "39* week*" or "40* week*" or "41* week*" or "42* week*" or "43* week*" or "44* week*"))):ti,ab,kw (Word variations have been searched)

#45 #36or#37or#38or#39or#40or#41or#42or#43or#44

#46 ((compar* near/10 gestation* near/3 week*)):ti,ab,kw (Word variations have been searched)

#47 ((compar* near/10 GW*)):ti,ab,kw (Word variations have been searched)

#48 #46 or #47

#49 #10 and #21 and #25

#50 #10 and #21 and #35

#51 #10 and #45

#52 #10 and #48

#53 #3or#49or#50or#51or#52

Appendix 2. List of studies and collaborators in OPTIMAL Collaborative Network

Study (year)	Sample Size	Country	Intervention timing	Ethnicity	Parity	BMI	Age	Socio-economic status	Assisted Conception
Studies agreed to share data (12,013 women)									
Place, 2023	381	Finland	41 weeks	NK	Yes	Yes	Yes	NK	NK
Keulen, 2019	1801	Netherlands	41 weeks	Yes	Yes	Yes	Yes	Yes	Yes
Wennerholm, 2019	2760	Sweden	41 weeks	Yes	Yes	Yes	Yes	Yes	Yes
Grobman, 2018	6106	USA	39 weeks	Yes	Yes	Yes	Yes	Yes	Yes
Walker, 2016	619	UK	39 weeks	Yes	Yes	Yes	Yes	NK	Yes
Yildrim, 2010	346	Turkey	38-40 weeks	NK	Yes	Yes	Yes	NK	NK
Studies contacted and awaiting response (4,262 women)									
Tan, 2021	159	Malaysia	39-40 weeks	Yes	Yes	Yes	Yes	Yes	NK
Sargunam, 2019	308	Malaysia	39 weeks	Yes	Yes	Yes	Yes	NK	NK
Zandvakili, 2019	151	Iran	39 weeks	NK	Yes	Yes	Yes	NK	NK
Baev, 2017	149	Russia	>40 weeks	NK	Yes	Yes	Yes	NK	NK
De Miranda, 2016	742	Netherlands	41 weeks	Yes	Yes	Yes	Yes	NK	NK
Miller, 2015	162	USA	>39 weeks	Yes	Yes	Yes	Yes	NK	NK
Brane, 2014	129	Sweden	37-41 weeks	Yes	Yes	Yes	Yes	Yes	NK
Ugwu, 2014	123	Nigeria	40-41 weeks	NK	Yes	Yes	Yes	NK	NK
BenitoReyes, 2010	200	Canary Islands	42 weeks	NK	Yes	Yes	Yes	NK	NK
Heimstad, 2007	508	Norway	41 weeks	Yes	Yes	Yes	Yes	NK	NK
Kashanian, 2006	101	Iran	39 weeks	NK	Yes	Yes	Yes	NK	NK
Gelisen, 2005	600	Turkey	41 weeks	NK	Yes	Yes	Yes	NK	NK
Sahraoui, 2005	150	France	41 weeks	NK	Yes	Yes	Yes	NK	NK
Nielsen, 2005	226	USA	39-40 weeks	Yes	Yes	Yes	Yes	NK	NK
Chanrachakul, 2003	249	Thailand	41 weeks	NK	Yes	Yes	Yes	NK	NK

James, 2001	74	India	41 weeks	NK	NK	NK	NK	NK	NK
Chakravarti, 2000	231	India	40-41 weeks	NK	NK	NK	NK	NK	NK
