# Supplementary material for: Optimal timing of induction of labour to improve maternal and perinatal outcomes: protocol for an individual participant data and network meta-analysis
Source: BMJ Open. 2026 Jan 8;16(1):e112155. doi: 10.1136/bmjopen-2025-112155 (PMC13059840; doi:10.1136/bmjopen-2025-112155)
Supplement: Supplementary file 2 [file bmjopen-2025-112155.reviewer_comments.pdf]

## PEER REVIEW HISTORY

BMJ Open publishes all reviews undertaken for accepted manuscripts. Reviewers are asked to complete a checklist review form and are provided with free text boxes to elaborate on their assessment. These free text comments are reproduced below.

### ARTICLE DETAILS

**Title (Provisional)**

Optimal timing of induction of labour to improve maternal and perinatal outcomes: protocol for an individual participant data and network meta-analysis

**Authors**

Meacham, Hollie; Ona-Igbru, Abraham; McNeill, Rachel; Ajayi, Ruth; Pickering, Emily; Grobman, William A; Black, Mairead; Khalil, Asma; Mccourt, Christine; Miranda, Angela; Mol, Ben W; Walker, Kate; Wilson, Amie; Zamora, Javier; Thangaratinam, Shakila; Allotey, John

---

This article was not externally reviewed at BMJ Open. Protocols that have been independently assessed prior to submission to BMJ Open are usually fast-tracked to publication on the grounds that further substantial changes will not be possible. This independent assessment will usually be external, independent review for both a substantial grant award from a non-commercial or government funder and ethics approval.
